# Risk factors for mortality among lung cancer patients with covid-19 infection: A systematic review and meta-analysis

**DOI:** 10.1371/journal.pone.0291178

**Published:** 2023-09-08

**Authors:** Mingyue Wu, Siru Liu, Changyu Wang, Yuxuan Wu, Jialin Liu

**Affiliations:** 1 Information Center, West China Hospital, Sichuan University, Chengdu, China; 2 Department of Biomedical Informatics, Vanderbilt University Medical Center, Nashville, TN, United States of America; 3 West China College of Stomatology, Sichuan University, Chengdu, China; 4 Department of Medical Informatics, West China Medical School, Sichuan University, Chengdu, China; 5 Department of Otolaryngology-Head and Neck Surgery, West China Hospital, Sichuan University, Chengdu, China; Korea Disease Control and Prevention Agency, REPUBLIC OF KOREA

## Abstract

**Background:**

Lung cancer patients with coronavirus disease 2019 (COVID-19) infection experience high mortality rates. The study aims to determine the risk factors for mortality in lung cancer patients with COVID-19 infection.

**Materials and methods:**

Followed the PRISMA reporting guidelines, PubMed, Embase, and Web of Science were systematically searched to February 20, 2023, for studies of lung cancer patients with COVID-19 infection. The main outcome of interest was the risk factor for mortality. We also compared the mortality rate of those patients among different continents. A pooled risk ratio (RR) with 95% CI was presented as the result of this meta-analysis.

**Results:**

Meta-analysis of 33 studies involving 5018 patients showed that pooled mortality rate of lung cancer in COVID-19 patients was 0.31 (95% CI: 0.25–0.36). Subgroup analysis based on the continents showed significant difference of the mortality rate was observed between Asia and the rest of world (*χ*^*2*^ = 98.96, *P* < 0.01). Older age (SMD: 0.24, 95% CI: 0.09–0.40, *P* < 0.01), advanced lung cancer (RR: 1.14, 95% CI: 1.04–1.26, *P* < 0.01), coexisting comorbidities such as hypertension (RR: 1.17, 95% CI: 1.01–1.35, *P* = 0.04) and cardiovascular disease (RR: 1.40, 95% CI: 1.03–1.91, *P* = 0.03) were associated with higher risk of mortality rate in those patients.

**Conclusions:**

Findings of this meta-analysis confirms an increased risk of mortality in lung cancer patients with COVID-19 infection, whose risk factors for these patients appear to be exacerbated by older age, advanced-stage lung cancer, and comorbidities such as hypertension and cardiovascular disease.

## Introduction

The coronavirus disease 2019 (COVID-19) outbreak, caused by the severe acute respiratory syndrome coronavirus 2 (SARS-CoV-2), has resulted more than 676 million confirmed cases worldwide as of March 10, 2023 [1–4]. This unprecedented crisis continues to challenge global public health systems as the number of new cases continues to rise. Compared to the general population, cancer patients are more susceptible to infections due to immunological impairment, either directly from the cancer itself or from immunosuppressive curative/palliative treatments [5–8].

Lung cancer remains the leading cause of cancer‐related death worldwide [9], and lung cancer patients are thought to be at a higher risk of severe COVID-19 complications [5, 10–12]. Evidence suggests that SARS-CoV-2 primarily infects the respiratory tract through the angiotensin-converting enzyme 2 (ACE2) receptor on alveolar epithelial cells, causing respiratory complications in the lung that may worsen lung cancer patients’ prognosis [13, 14]. Numerous studies have attempted to determine the outcomes of lung cancer patients with COVID-19 infection to aid in risk stratification and clinical management [8, 11, 12, 15–53]. In a recent multicenter cohort study conducted by Mengyuan Dai [8], it was found that patients with hematologic cancer, lung cancer, or metastatic cancer (stage IV) had the highest frequency of severe events. This supports the findings by Garassino [11] that patients with thoracic cancer have a high mortality rate. However, there is a lack of meta-analyses specifically focused on patients with lung cancer and COVID-19. The existing published studies have mainly analyzed whether lung cancer is a poor prognostic factor for COVID-19 [54–56]. Oldani [54] compared the disease severity and mortality rate between lung cancer patients and non-cancer patients, as well as patients with other malignancies. The results showed that the mortality rate of lung cancer patients was significantly higher than that of non-cancer patients or patients with other malignancies. However, it was not statistically significant to consider lung cancer as a risk factor for SARS-CoV-2 infection. In the overall meta-analysis conducted by Haike Lei [55], no significant differences in mortality rates were found between lung cancer and other tumor subgroups. This suggests that there may be other potential risk factors for lung cancer patients with COVID-19, which could be the underlying causes of the increased mortality.

Therefore, the specific risk factors that contribute to the higher mortality rate of lung cancer patients with COVID-19 remain uncertain. Additionally, there is a lack of meta-analysis reports on this topic. Thus, the objective of our study is to identify the risk factors associated with mortality in lung cancer patients who have contracted COVID-19, by analyzing the existing published data. We anticipate that the findings from this meta-analysis will offer significant insights for safeguarding individuals who are vulnerable to COVID-19.

## Materials and methods

### Study protocol

In accordance with the Preferred Reporting Items for Systematic Reviews and Meta-analyses (PRISMA) reporting guidelines [57], this study protocol has been registered in the International Prospective Registry of Systematic Reviews (PROSPERO) (number: CRD42022306866) and published in *PLOS ONE* [58].

### Search strategy and literature search

We performed repeated searches of PubMed, Embase, and Web of Science for English-language articles published up to February 20, 2023. We identified relevant articles from the references of these articles and removed duplicates. The search strategy included the following terms: ("COVID-19" or " SARS-CoV-2" or "coronavirus disease 2019" or "2019-nCoV") and ("cancer" or "neoplasms" or "carcinoma" or "malignancy") and ("lung" or "pulmonary"). Details of the results of the literature search can be found in S1 Table in the supplementary material.

### Study selection

Two authors (M.W. and S.L.) independently screened all titles and abstracts after the initial search, and discrepancies were resolved by consultation with a third author (J.L.).

Inclusion criteria were as follows: (1) any cohort studies, case-control, and cross-sectional studies; (2) lung cancer patients with COVID-19 infection confirmed through Reverse Transcriptase Polymerase Chain Reaction (RT-PCR) or highly suspected COVID-19 infection identified by CT scan or symptoms, to ensure data integrity; and (3) studies reporting clinical characteristics or outcomes.

Exclusion criteria were as follows: (1) publication types such as abstracts, editorials, letters, reviews, commentaries, guidelines, or non-human studies; (2) studies with data that could not be obtained from the corresponding author; and (3) repetitive data published in the literature.

### Data extraction

Two authors (M.W. and S.L.) independently performed data extraction and quality assessment, and cross-checked the extracted data to reach consensus. The extraction included: (1) study information: first author, single or multi-center study type (retrospective or prospective), study period, study region, diagnostic methods of COVID-19, and study sample; (2) demographic or clinical characteristics: age, sex, smoking history (former/current smoker vs. non-smoker), lung cancer type (NSCLC vs. other), lung cancer stage (advanced stage or other), laboratory parameters, common COVID-19 symptoms, and comorbidities; and (3) outcomes: death status of lung cancer patients. Advanced cancer included stage III/IV or metastasis, regardless of whether the cancer type was NSCLC or other.

### Statistical analysis

We assessed the quality of the included articles using the Newcastle-Ottawa Quality Assessment Scale (NOS) for case-control and cohort studies [59], and the Prevalence Study Quality for cross-sectional studies [60]. We assessed publication bias between studies by visually inspecting a funnel plot for asymmetry and performing Egger’s test (*P* < 0.05 was considered to indicate significant publication bias) [61].

The main outcome of interest was the risk factor for mortality in lung cancer patients with COVID-19 infection. Therefore, we compared the collected demographic or clinical characteristics mentioned above between living and dead lung cancer patients with confirmed COVID-19 infection. We also compared the mortality of lung cancer patients with COVID-19 infection between different continents. Qualitative results of this meta-analysis were presented as pooled risk ratios (RRs) with 95% confidence intervals (CIs), and quantitative results were presented as standard mean differences (SMD) with 95% CIs.

Statistical heterogeneity was quantified using the I^2^ statistic [62]. The common-effects model was used when the heterogeneity was not significant (*P* ≥ 0.05, I^2^ ≤ 50%), whereas the random-effects model was performed when heterogeneity between studies was present (*P* < 0.05, I^2^ > 50%) [63]. For the confounding variables, we have incorporated six factors to examine the influence of these variables on the study. These factors include the center type, continents, publication year, study design, study type, and diagnosis methods for COVID-19. We have employed meta-regression analysis to ascertain if these confounding factors have a noteworthy impact on the established effect model. Subgroup analysis was then performed to dissect significant heterogeneity for those variables with a *P* value less than 0.05. Sensitivity analysis was also used to assess the stability of the results by iteratively repeating the analyses, discarding one dataset each time.

Meta-analysis was performed using R software (version 4.1.2). A two-sided *P* < 0.05 indicated statistical significance.

## Results

### Literature search results

**Fig 1:**
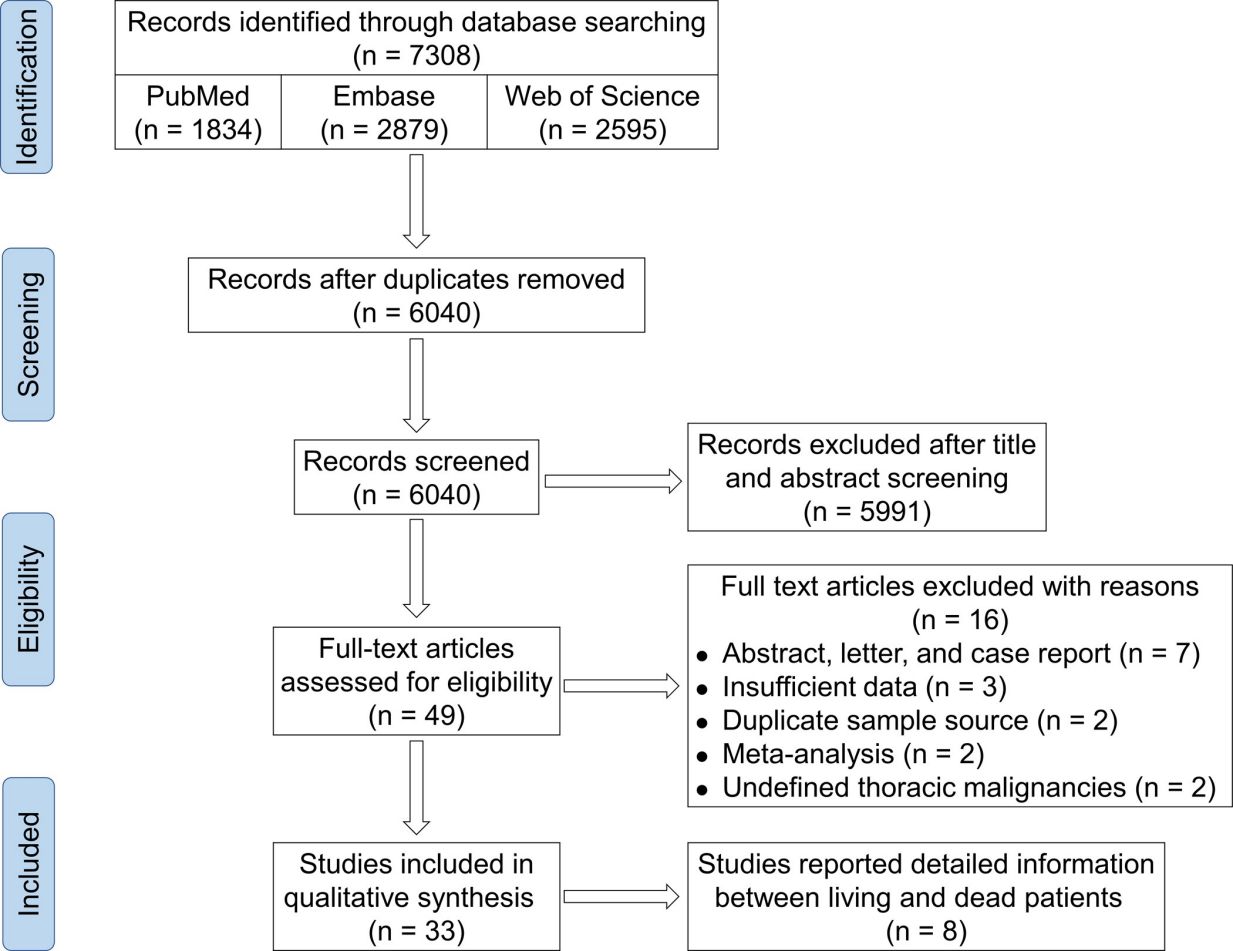
Details of the PRISMA chart for study selection.

A total of 7,308 articles were initially retrieved for this study. After removing irrelevant or duplicate articles, 6,040 records remained for title and abstract screening. Of these, we selected 49 articles for full-text screening. To avoid duplication of the population, we retained studies with the largest number of samples for duplicate sample sources. Ultimately, according to the pre-specified inclusion criteria, 33 studies remained for this systematic review and meta-analysis, including 8 studies that reported detailed information between living and dead lung cancer patients with COVID-19 infection. Details of the PRISMA chart for study selection are shown in Fig 1.

### Global distribution of studies

**Fig 2:**
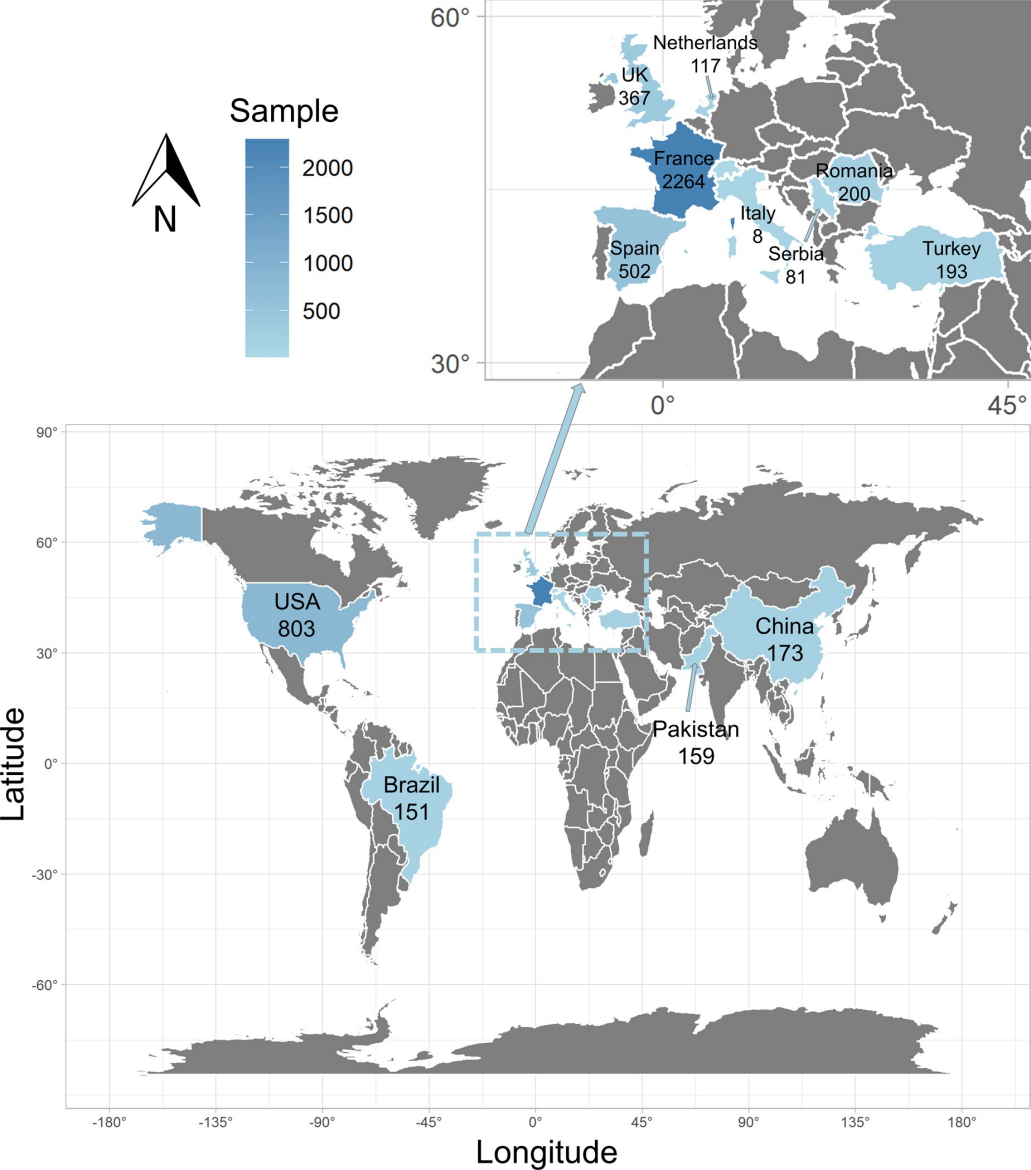
The distribution of lung cancer patients across 33 studies.

The 33 studies included 5,018 patients with lung cancer and COVID-19 infection from 12 countries on 4 continents. Europe had the highest number of patients recruited (n = 3,539, 70.53%), followed by North America (n = 803, 16.00%), Asia (n = 525, 10.46%), and South America (n = 151, 3.01%). The distribution map of lung cancer patients across the 33 studies is shown in Fig 2, which was generated using the ggplot2 package in the R software program.

### Characteristics of included studies

Basic information about the 33 included studies is summarized in Table 1. These studies consisted of 25 retrospective, 7 prospective, and 1 retro-prospective study. More than half of the included studies were multi-center studies (n = 21, 63.64%). The diagnosis of patients with COVID-19 infection was mainly confirmed by RT-PCR. The overall quality of the 33 studies was high, with quality scores ranging from 5 to 9 (S2 and S3 Tables) (S1 Fig). The funnel plot is shown in S2 Fig, and Egger’s test (t = - 0.19, *P* = 0.85) indicated no significant publication bias among the included studies.

**Table 1:** Characteristics of the included 33 studies.

Author/Year	Region	Lung Cancer Patients with COVID-19		Single/Multi-center	Study design	Study type	Diagnosis method for Covid-19	Quality Score
		Total (n = 5018)	Deaths (n = 1624)					
Beltramo et al., 2021 [40]	France	977	402	Multi-center	Retrospective	Cohort study	RT-PCR	6
Bernard et al., 2021 [41]	France	873	359	Multi-center	Retrospective	Cohort study	RT-PCR	6
Chen et al., 2022 [42]	USA	676	63	Multi-center	Prospective	Cohort study	RT-PCR	6
Provencio et al., 2021 [15]	Spain	447	146	Multi-center	Prospective	Cohort study	RT-PCR	5
Lièvre et al.,2020 [43]	France	311	113	Multi-center	Retro-prospective	Cohort study	RT-PCR/CT/Clinical	7
Várnai et al.,2022 [44]	UK	265	131	Multi-center	Prospective	Cohort study	RT-PCR	6
Haineala et al., 2021 [22]	Romania	200	64	Multi center	Prospective	Cohort study	RT-PCR/CT/Clinical	7
Farooque et al., 2021 [45]	Pakistan	159	7	Multi-center	Prospective	Cohort study	RT-PCR	8
Özdemir et al.,2020 [46]	Turkey	157	18	Multi-center	Retrospective	Cohort study	RT-PCR	7
Joode et al., 2022 [53]	Netherlands	117	44	Multi-center	Retrospective	Cohort study	RT-PCR/Antibodies	8
Peixoto et al., 2022 [27]	Brazil	110	58	Single	Retrospective	Cohort study	RT-PCR/CT/Clinical	7
Luo et al., 2020 [19]	USA	102	25	Single	Retrospective	Cohort study	RT-PCR	7
Lee et al.,2020 [47]	UK	90	32	Multi-center	Retrospective	Cohort study	RT-PCR	6
Benderra et al., 2021 [48]	France	85	37	Multi-center	Retrospective	Cohort study	RT-PCR	5
Bursać et al., 2022 [24]	Serbia	81	16	Single	Retrospective	Case control study	RT-PCR	7
Song et al.,2021 [49]	China	61	16	Multi-center	Retrospective	Cohort study	RT-PCR	6
Nie et al., 2021 [16]	China	45	11	Multi-center	Retrospective	Cohort study	RT-PCR	6
Kahya et al., 2021 [18]	Turkey	36	2	Single	Retrospective	Case control study	RT-PCR	8^#^
Calles et al., 2020 [25]	Spain	23	8	Single	Retrospective	Cross-sectional study	RT-PCR	6^#^
Dai et at., 2020 [8]	China	22	4	Multi-center	Retrospective	Case control study	RT-PCR	8
Zhang et al., 2020 [31]	China	21	5	Multi-center	Retrospective	Cross-sectional study	RT-PCR	8^#^
Basse et al., 2021 [36]	France	18	6	Single	Prospective	Cohort study	RT-PCR/CT	8
Fernandes et al., 2021 [50]	Brazil	18	6	Single	Retrospective	Cross-sectional study	RT-PCR	7^#^
Rogado et al., 2020 [17]	Spain	17	9	Single	Retrospective	Cohort study	RT-PCR	7
Ferrari et al., 2021 [51]	Brazil	16	7	Multi-center	Prospective	Cohort study	RT-PCR	6
Yarza et at., 2020 [32]	Spain	15	6	Single	Retrospective	Cohort study	RT-PCR/CT/Clinical	7
Yang et al.,2020 [33]	China	24	6	Multi-center	Retrospective	Cohort study	RT-PCR	7
Khusid et al.,2021 [52]	USA	14	6	Multi-center	Retrospective	Cohort study	RT-PCR	6
Mehta et al.,2020 [35]	USA	11	6	Single	Retrospective	Cohort study	RT-PCR	7
Stroppa et al.,2020 [37]	Italy	8	2	Single	Retrospective	Cohort study	RT-PCR	6
Fraser et al., 2021 [29]	UK	7	2	Multi-center	Retrospective	Cohort study	RT-PCR	6
de Melo et al., 2020 [34]	Brazil	7	4	Single	Retrospective	Cohort study	RT-PCR	7
Hogan et al., 2020 [38]	UK	5	3	Multi-center	Retrospective	Cohort study	RT-PCR	7

^#^ Quality of studies was evaluated through Prevalence Study Quality for cross-sectional studies, while others by Newcastle-Ottawa quality assessment scale.

### The mortality of lung cancer patients with COVID-19 infection

All included studies reported the mortality of lung cancer patients with COVID-19 infection, resulting in 1,624 deaths among 5,018 patients. The mortality varied among different regions, with the highest in Brazil (75/151, 49.67%) and the lowest in Pakistan (7/159, 4.40%) (S3 Fig). The pooled mortality of lung cancer in COVID-19 patients was 0.31 (95% CI: 0.25–0.36) (S4 Fig). Sensitivity analysis revealed that our results were robust (S5 Fig). However, our analysis showed that the heterogeneity between studies was substantial (I^2^ = 94%, *P* < 0.01). The result of the meta-regression analysis showed that there was significant heterogeneity in the continents of the included studies (*Z* = 4.93, *P* < 0.01) (S4 Table).

**Fig 3:**
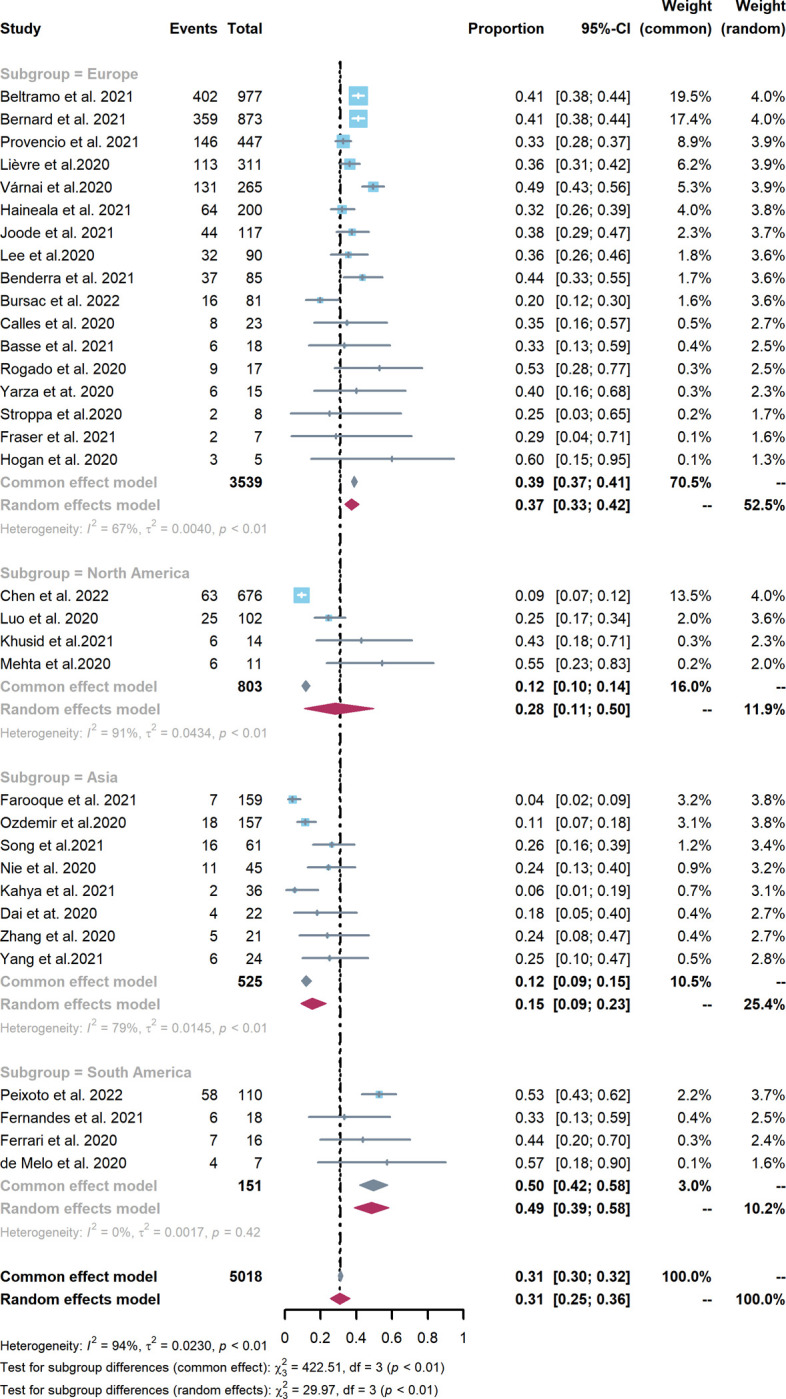
Pooled mortality of lung cancer in COVID-19 patients after subgroup analysis based on the continents.

As shown in Fig 3, subgroup analysis by continent revealed that the pooled lung cancer mortality in COVID-19 patients was 0.50 (95% CI: 0.42–0.58) in South America, followed by Europe [0.37 (95% CI: 0.33–0.42)], North America [0.28 (95% CI: 0.11–0.50)], and Asia [0.15 (95% CI: 0.09–0.23)]. A significant difference in mortality was observed between Asia and the rest of the world (*χ*^*2*^ = 98.96, *P* < 0.01). However, the source of heterogeneity could not be further determined due to limited information in the 33 studies.

### Risk factor for mortality in lung cancer patients with COVID-19 infection

Eight of the 33 studies (24.24%), including 260 deaths among 771 patients, reported detailed information between living and dead lung cancer patients with confirmed COVID-19 infection by RT-PCR. Considering that these studies reported different demographic or clinical characteristics, the corresponding data were scrutinized, and insufficient information reported by less than 3 studies was excluded. Finally, age, sex, smoking history, lung cancer type, lung cancer stage, and comorbidities, including hypertension, cardiovascular disease, respiratory disease, and diabetes mellitus, were selected to assess the risk factors for mortality (Table 2).

**Table 2:** The detailed information for lung cancer patients with COVID-19 infection.

Group	Study	Sample	Age	Gender		Smoking status		Cancer type		Cancer stage		Comorbidities			
				Male	Female	Former/Current	Non-smoker	NSCLC	Others	Advanced-stage	Others	Hypertension	Cardiovascular disease	Respiratory disease	Diabetes mellitus
**Dead**	Provencio et al., 2021 [15]	146	68.00 ± 9.40	108	38	128	18	120	26	125	21	73	45	42	27
	Peixoto et al., 2022 [27]	58	67.00 ± 8.00	32	26	46	12	53	5	40	18	28	NR	19	22
	Luo et al., 2020 [19]	25	71.79 ± 13.36	14	11	NR	NR	NR	NR	15	10	18	6	18	9
	Nie et al., 2020 [16]	11	72.57 ± 11.03	10	1	3	8	5	6	9	2	7	3	NR	3
	Dai et at., 2020 [8]	4	67.75 ±13.12	2	2	2	2	1	3	2	2	2	NR	NR	1
	Zhang et al., 2020 [31]	5	70.60 ± 8.50	4	1	2	3	NR	NR	3	2	3	1	2	2
	Rogado et al., 2020 [17]	9	64.87 ± 11.38	6	3	NR	NR	NR	NR	7	2	6	NR	4	NR
	Fraser et al., 2021 [29]	2	64.35 ± 5.94	2	0	1	1	NR	NR	NR	NR	2	0	0	1
**Living**	Provencio et al., 2021 [15]	301	66.60 ± 9.90	224	77	255	46	257	44	229	72	134	69	95	75
	Peixoto et al., 2022 [27]	52	63.00 ± 10.00	24	28	41	11	48	4	26	26	29	NR	17	19
	Luo et al., 2020 [19]	77	67.30 ± 9.07	35	42	NR	NR	NR	NR	53	24	39	1	34	18
	Nie et al., 2020 [16]	34	63.49 ± 11.61	21	13	8	26	10	24	22	12	8	4	NR	3
	Dai et at., 2020 [8]	18	66.78 ± 6.83	16	2	12	6	9	9	12	6	3	2	1	4
	Zhang et al., 2020 [31]	16	67.00 ± 6.11	12	4	3	13	NR	NR	7	9	8	1	2	2
	Rogado et al., 2020 [17]	8	69.26 ± 11.85	7	1	NR	NR	NR	NR	4	4	4	NR	5	NR
	Fraser et al., 2021 [29]	5	74.18 ± 7.21	3	2	4	1	NR	NR	NR	NR	5	0	1	0

Age was presented as mean and standard deviation, and others were the number of patients

NR, Not reported; NSCLC, non-small cell lung cancer; advanced-stage cancer including stage III/IV or metastasis

**Fig 4:**
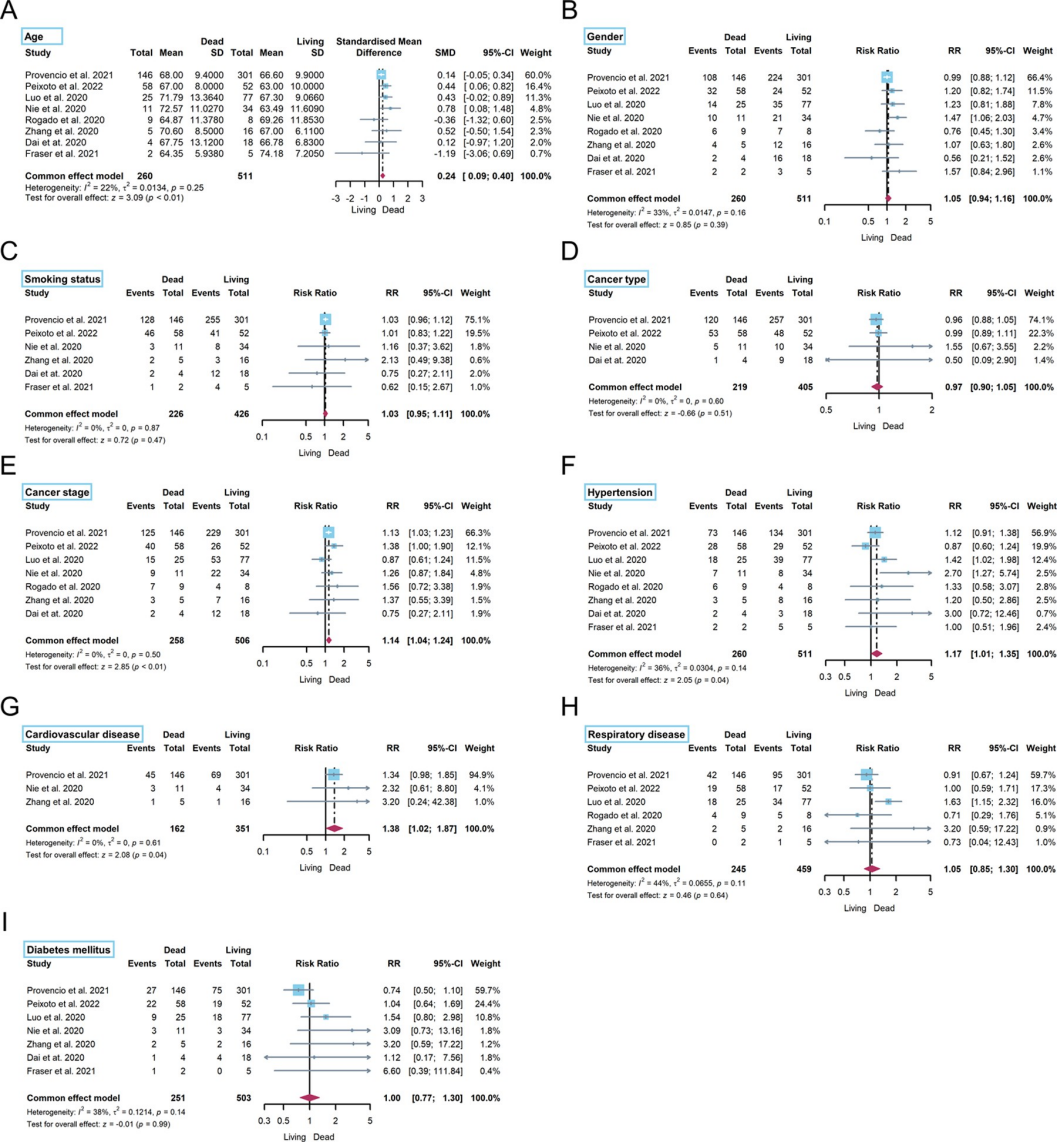
Forest plot for risk factors of case mortality of lung cancer patients with COVID-19 infection.

As shown in Fig 4, eight studies provided data on the age of living and dead lung cancer patients with COVID-19 infection. The result showed that older patients had a higher risk of death (SMD: 0.24, 95% CI: 0.09–0.40, *P* < 0.01). Seven studies reported the cancer stage information, and the result demonstrated that advanced-stage lung cancer including stage III/IV or metastasis was also associated with a higher risk of death compared with others (RR: 1.14, 95% CI: 1.04–1.26, *P* < 0.01). In term of comorbidities, there were significant differences in hypertension (RR: 1.17, 95% CI: 1.01–1.35, *P* = 0.04) between living and dead lung cancer patients with COVID-19 infection. As for cardiovascular disease reported by four studies, an apparent asymmetry was observed when assessing for publication bias (S6 Fig). Thus, a sensitivity analysis was performed using the leave-one-out approach and a significant change in the result was observed, showing that cardiovascular disease was also associated with an increased rate of death (RR: 1.40, 95% CI: 1.03–1.91, *P* = 0.03). However, the results showed that sex, smoking history, lung cancer type, respiratory disease, and diabetes mellitus were not associated with a higher risk of death (*P* = 0.39, 0.47, 0.51, 0.64, 0.99, respectively).

## Discussion

A major strength of our study is the detailed investigation of risk factors for mortality in lung cancer patients with COVID-19 infection. Our findings indicate that older age, advanced-stage lung cancer, and comorbidities such as hypertension and cardiovascular disease contribute to increased mortality in this patient population. These observations align with previous research on risk factors for COVID-19-related mortality in the general population [64, 65]. Interestingly, our study did not identify any significant associations between gender, smoking history, lung cancer subtype, respiratory disease, and diabetes mellitus and the risk of death in lung cancer patients with COVID-19 infection.

According to the results of two previous meta-analyses on COVID-19 and cancer, cancer was associated with an increased risk of severe COVID-19 or death compared to patients without cancer [66, 67]. Considering that the lung is a primary target of SARS-CoV-2, and given the inherent vulnerability of lung cancer patients [5–8, 10–12], three previous meta-analyses sought to determine whether lung cancer was a poor prognostic factor for COVID-19 [54–56]. However, these studies varied in size and methodology, produced inconsistent results when assessing the differences in mortality between lung cancer patients and those with other malignancies and COVID-19 infection. Moreover, these meta-analyses were limited by the absence of detailed information comparing lung cancer patients who survived and those who succumbed to COVID-19 infection. Thus, it remains unclear which risk factors would increase mortality in lung cancer patients with COVID-19 infection.

A strength of our study is the comprehensive systematic review of 33 individual studies with a wide geographical distribution to represent the global impact on lung cancer patients with COVID-19 infection. Our results indicate that the pooled mortality in lung cancer patients with COVID-19 infection is estimated to be 0.31 (95% CI: 0.25–0.36), which is higher than that observed in the general population with COVID-19 infection as reported in recently published studies [1, 35, 68, 69]. Furthermore, our continent-based subgroup analysis shows that the pooled lung cancer mortality in COVID-19 patients is 0.15 (95% CI: 0.09–0.23) in Asia, which is significantly lower than the rest of the world (*χ*^*2*^ = 98.96, *P* < 0.01). This disparity is mainly due to the different approaches to handling the SARS-CoV-2 pandemic in different regions and countries, where health policies and lifestyles may have influenced mortality rates from lung cancer or COVID-19 infection. Nevertheless, the results of our study underscore the need for continued data sharing and international collaboration on a global scale.

Another crucial issue addressed in our study is to the underlying risk factors associated with COVID-19-related mortality in lung cancer patients. In particular, recent studies have identified older age and smoking as the most significant risk factors for lung cancer [64, 65, 70]. Other risk factors include air pollution, previous radiotherapy, respiratory disease, and family history [70, 71]. Our systematic review and meta-analysis support the hypothesis that older age, advanced-stage lung cancer, and comorbidities such as hypertension and cardiovascular disease are associated with a higher risk mortality in lung cancer patients with COVID-19 infection. Previously, older age was reported to be an important risk factor for mortality in SARS-CoV-2 infection. Additionally, age-dependent defects in B-cell and T-cell function could result in prolonged proinflammatory responses and a deficiency in controlling viral replication, potentially leading to poor outcomes [72]. Given that older age is also a risk factor for lung cancer[65], special treatment should be considered for these patients. Patients with advanced-stage lung cancer often have decreased immune function, reduced lung function, and severe infections, which may contribute to worse outcomes in this subpopulation. Other risk factors related to mortality include comorbidities such as hypertension and cardiovascular disease. Crackower et al.’s evidence [73] demonstrated that SARS-CoV-2 could directly impair cardiac function by targeting disruption of ACE2 in mice, resulting in severe cardiac contractility defects, increased angiotensin II levels, and upregulation of hypoxia-induced genes in the heart. Previous studies have also shown that circulating ACE2 levels are higher in patients with hypertension and cardiovascular disease [74, 75]. As a result, lung cancer patients with these comorbidities may have a potentially worse prognosis and an increased risk of death from COVID-19 infection due to high expression of the ACE2 receptor. Based on these findings, we recommend that high-risk lung cancer patients be evaluated and treated in a clinical setting with comprehensive medical resources.

This study has several limitations. Firstly, demographic or clinical characteristics were only reported in 8 of the 33 individual studies (24.24%). Additionally, the lack of more detailed information on anticancer treatment may impact the baseline prognosis of lung cancer patients with COVID-19 infection. Second, the potential mortality of SARS-CoV-2 variants could not be examined, as this information was not available. Thirdly, the source of heterogeneity could not be further determined when evaluating the pooled mortality of the 33 studies due to limited information. However, no significant heterogeneity was found when assessing the risk factor for mortality using the leave-one-out approach of the sensitivity analysis. Finally, due to the nature of retrospective studies and only including articles written in English, it was not possible to completely avoid any potential bias in selecting the articles for this study.

Thus, this systematic review and meta-analysis brought attention to possible directions for future investigation, such as (1) conducting additional prospective and multi-center cohort studies involving diverse regions and languages; (2) gathering detailed data on the systemic therapy employed for cancer treatment; (3) exploring the potential impact of different variants of the SARS-CoV-2 virus on mortality. Additionally, to ascertain the effectiveness of nationwide vaccination campaigns, it is important for future studies to assess COVID-19 mortality rates among lung cancer patients who have been vaccinated. It would also be valuable to analyze the impact of vaccine dosage and type on the antibody response.

## Conclusions

Based on our meta-analysis, we have found that there is an increased mortality risk in lung cancer patients with COVID-19. The risk factors for these patients seem to be amplified by older age, advanced lung cancer, and comorbidities such as hypertension and cardiovascular disease. Implementing evidence-based best practices will help mitigate the impact of the COVID-19 pandemic on lung cancer patients and reduce the risk of death from COVID-19 infection.

## Supporting information

S1 Fig Heatmap of quality assessment of the included studies. (TIF)

S2 Fig Funnel plot and egger’s test showed no publication bias among the included 33 studies (*P* = 0.85). (TIF)

S3 Fig Mortality of lung cancer patients with COVID-19 infection among different countries. (TIF)

S4 Fig The pooled mortality of lung cancer in COVID-19 patients. (TIF)

S5 Fig Sensitivity analysis of lung cancer mortality in COVID-19 patients. (TIF)

S6 Fig Funnel plot for risk factors of case mortality of lung cancer patients with COVID-19 infection. (TIF)

S1 Table Search strategy used in PubMed, Embase, and Web of Science. (DOCX)

S2 Table Quality assessment of the included studies by the Newcastle-Ottawa scale. The maximal score for Newcastle-Ottawa scale is 9 stars: 4 stars for the selection process, 2 stars for comparability, and 3 stars for outcome.
(DOCX)

S3 Table Quality assessment of the included studies by the Prevalence Study Quality. The maximal score for Prevalence Study Quality is 11, and only *YES* means 1 score.
(DOCX)

S4 Table The result of meta-regression analysis when evaluating the pooled mortality of lung cancer in COVID-19 patients. ^#^ variables were set as the reference. *P value < 0.05.
(DOCX)

S5 Table PRISMA checklist. (DOCX)
