# Supplementary figures and images for: Risk factors for mortality among lung cancer patients with covid-19 infection: A systematic review and meta-analysis
Source: PLoS One. 2023 Sep 8;18(9):e0291178. doi: 10.1371/journal.pone.0291178 (PMC10490932; doi:10.1371/journal.pone.0291178)

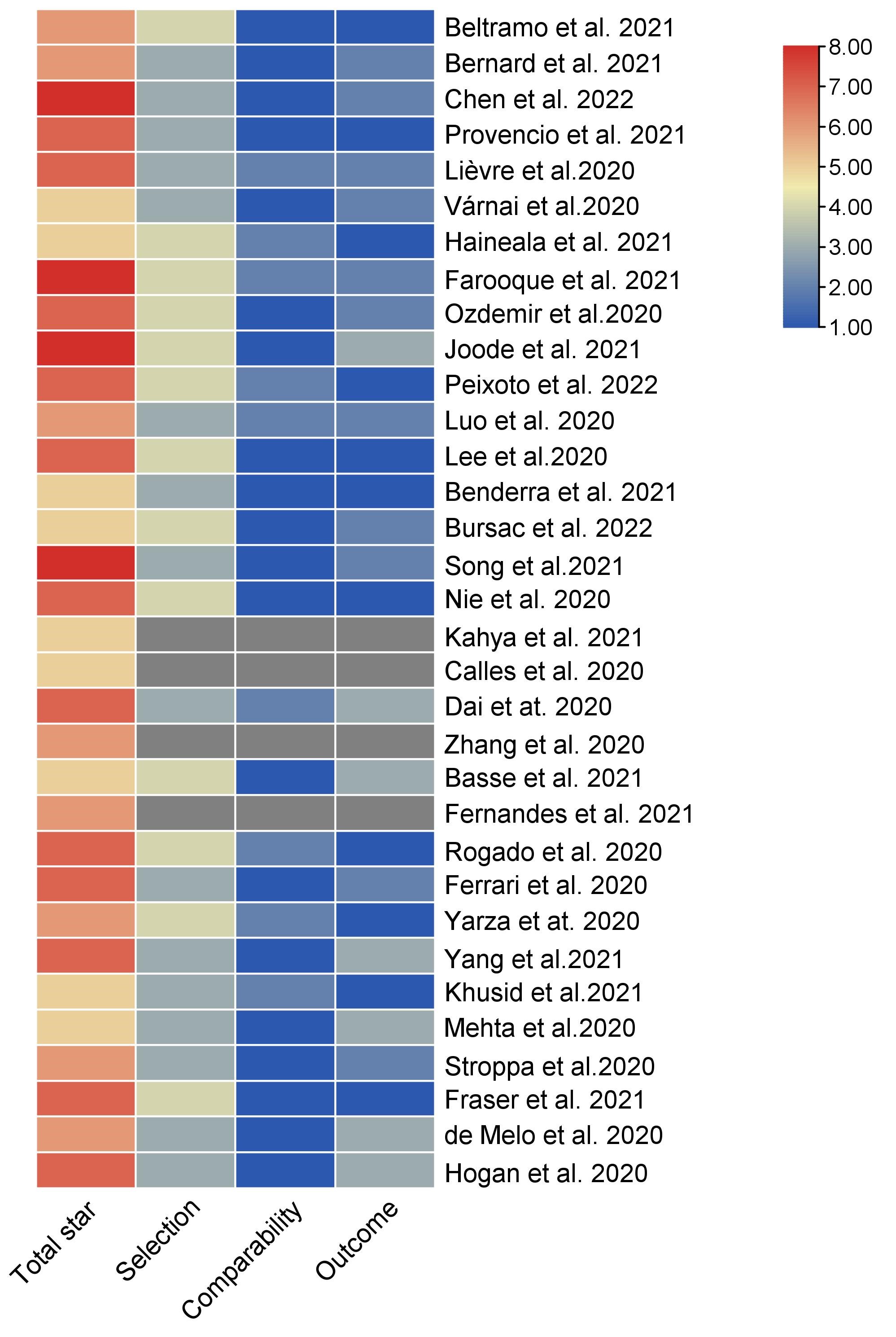

Supplement: S1 Fig — (TIF) [file pone.0291178.s001.tif]

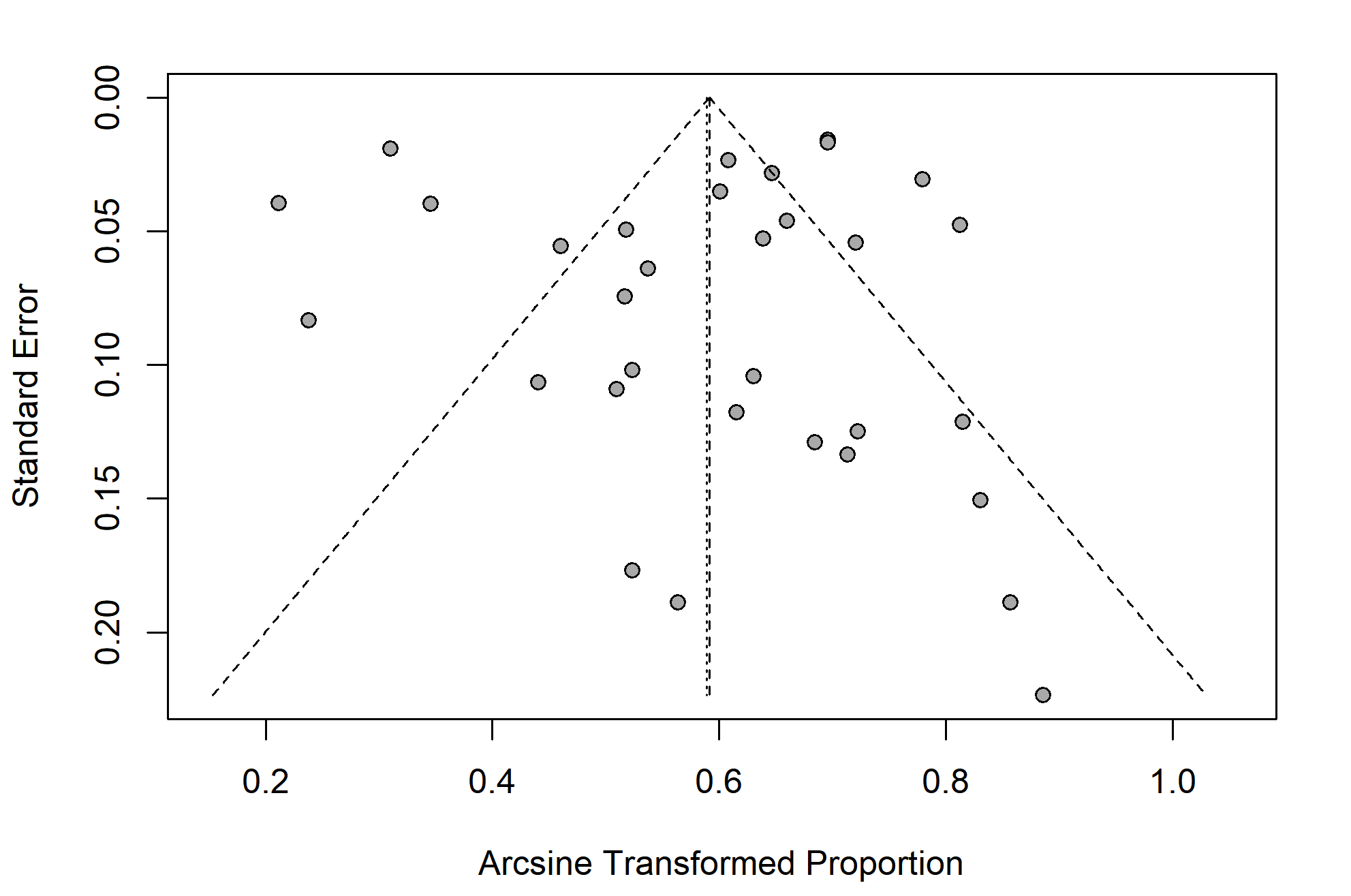

Supplement: S2 Fig — (TIF) [file pone.0291178.s002.tif]

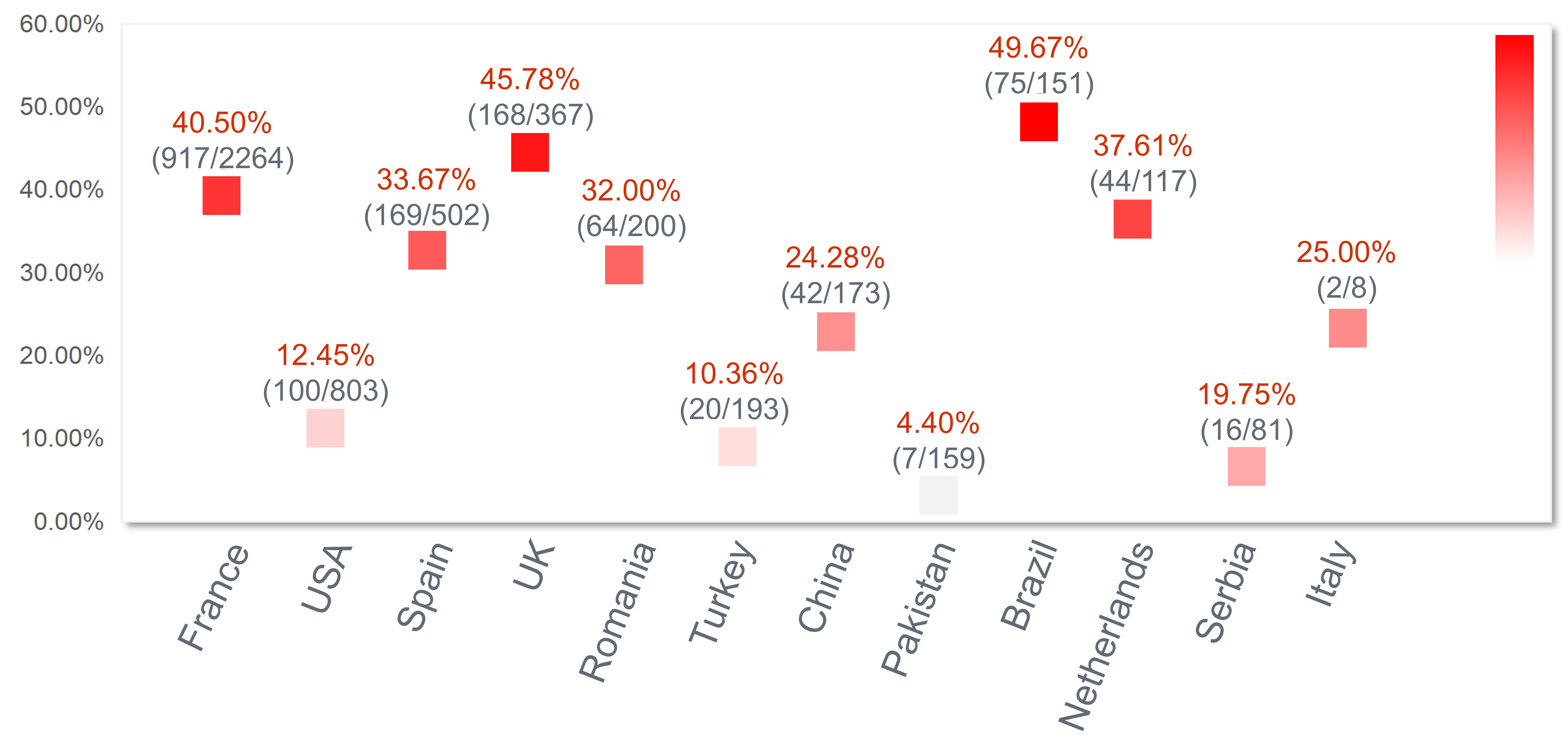

Supplement: S3 Fig — (TIF) [file pone.0291178.s003.tif]

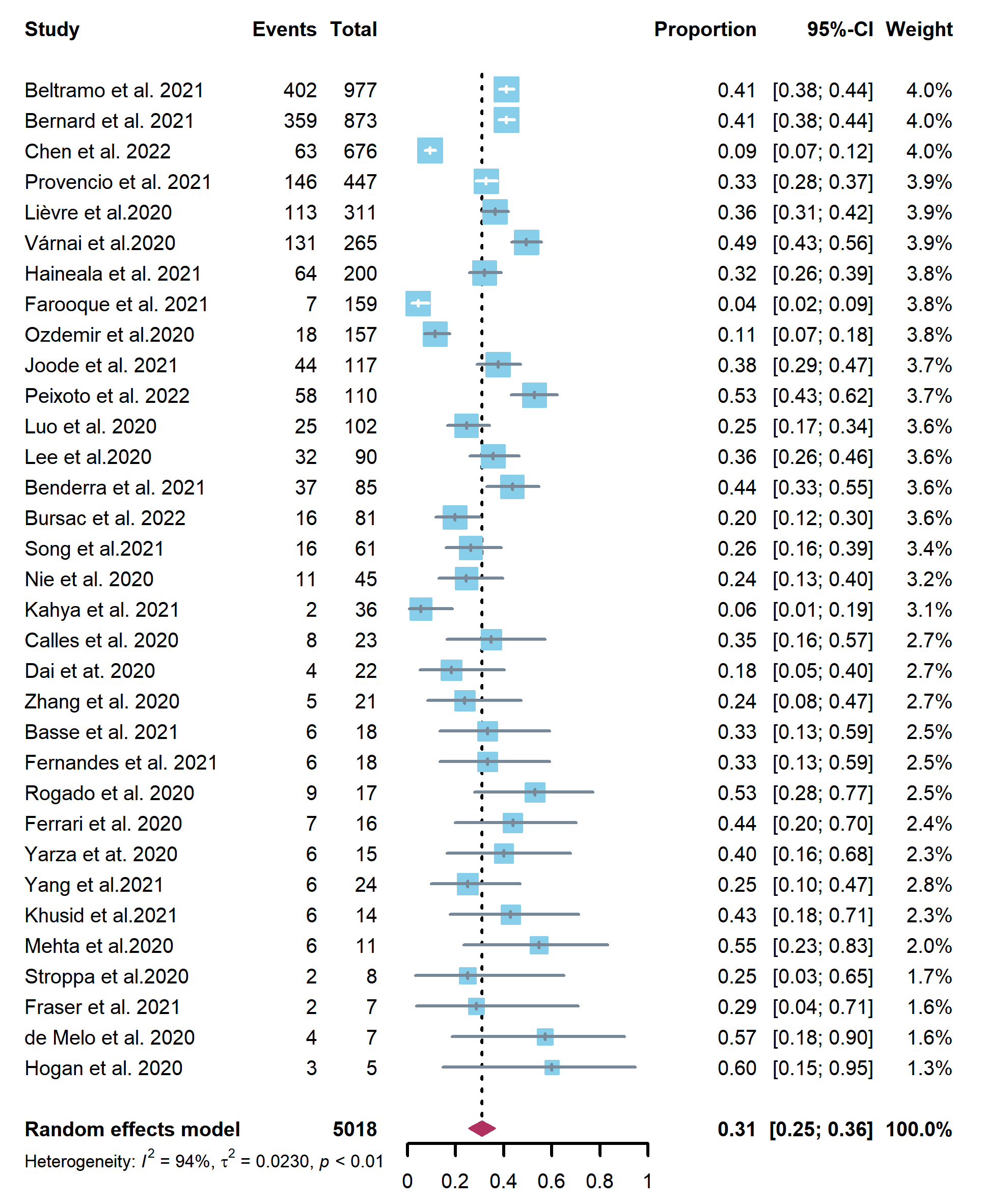

Supplement: S4 Fig — (TIF) [file pone.0291178.s004.tif]

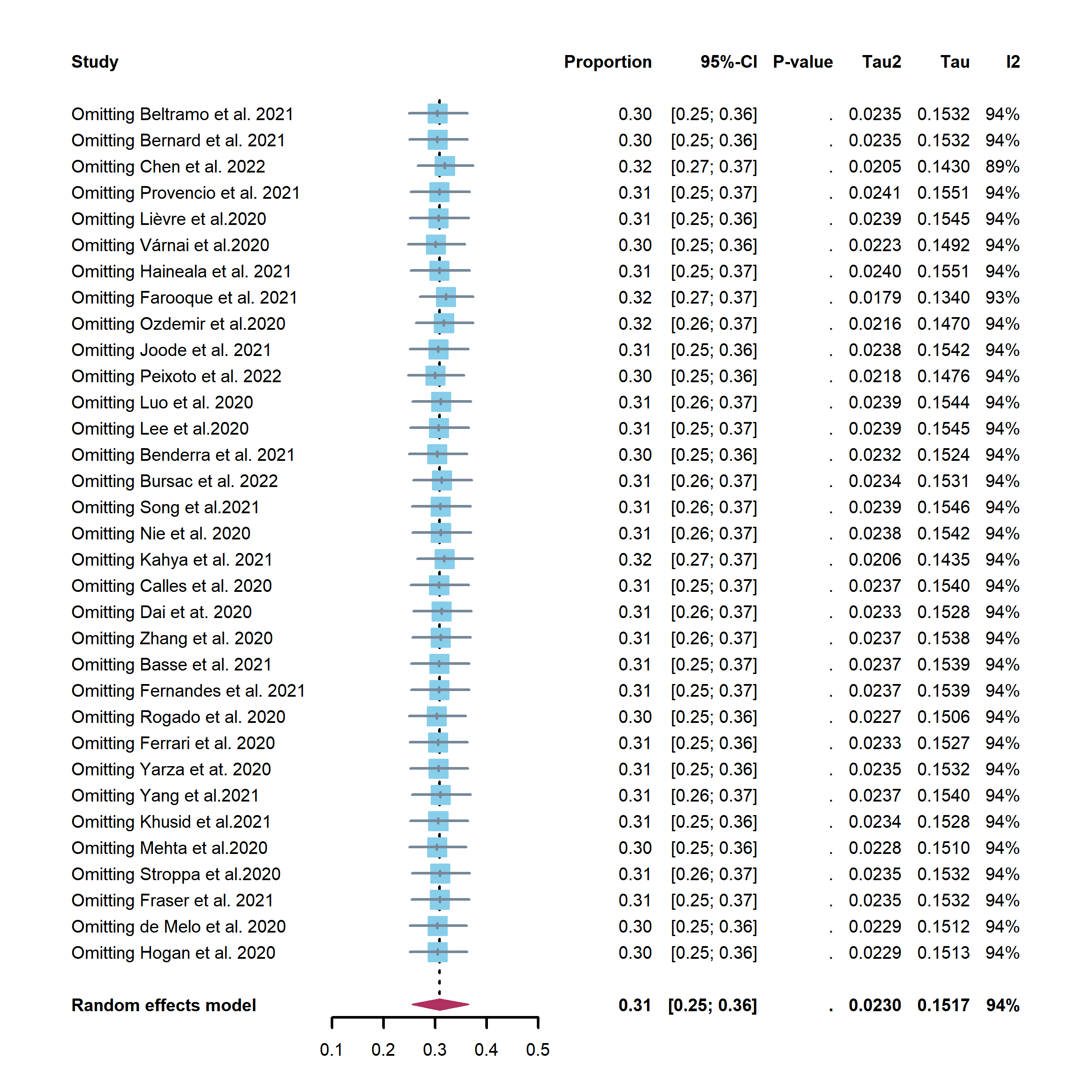

Supplement: S5 Fig — (TIF) [file pone.0291178.s005.tif]

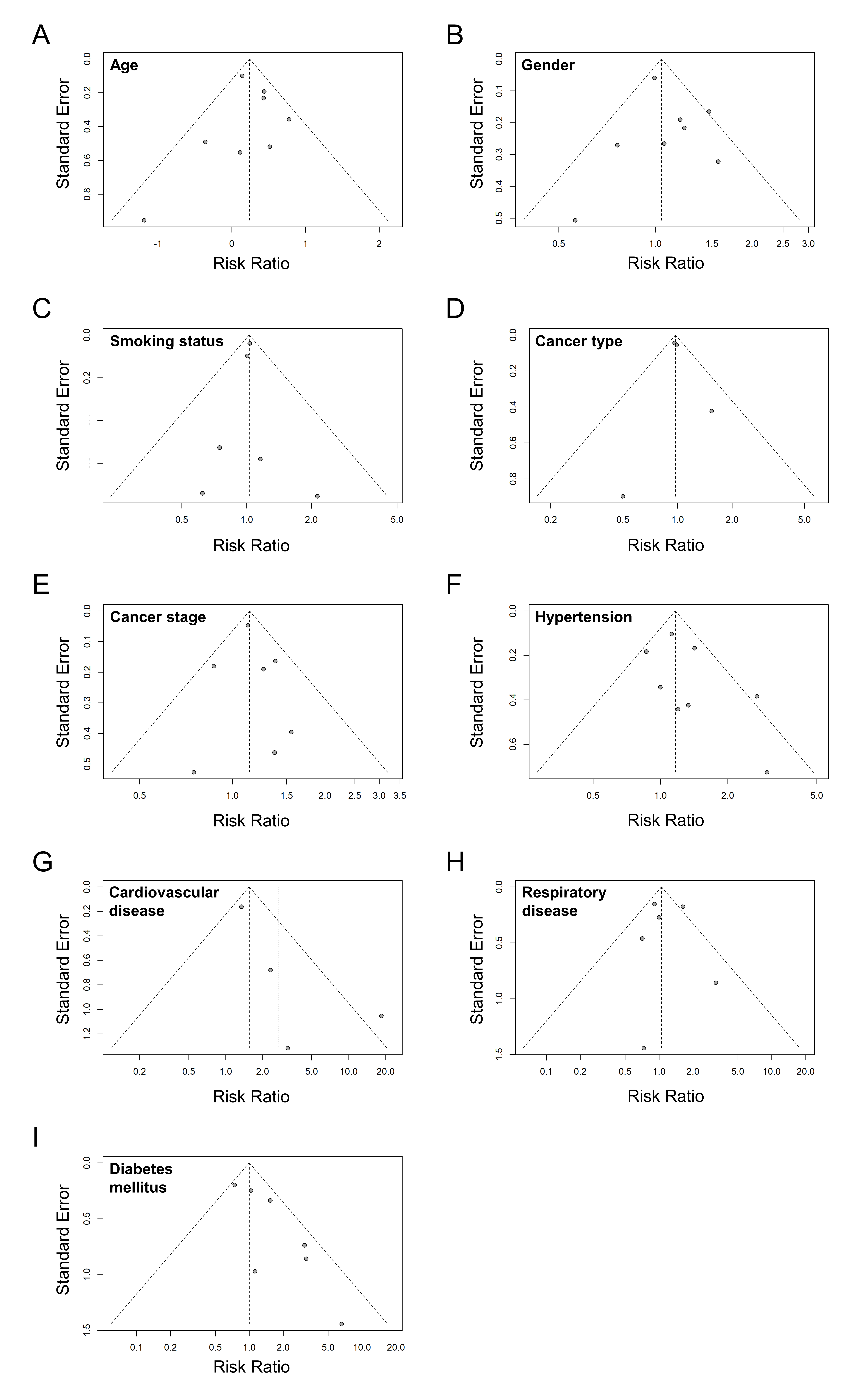

Supplement: S6 Fig — (TIF) [file pone.0291178.s006.tif]
