# Supplementary material for: Risk factors for mortality among lung cancer patients with covid-19 infection: A systematic review and meta-analysis
Source: PLoS One. 2023 Sep 8;18(9):e0291178. doi: 10.1371/journal.pone.0291178 (PMC10490932; doi:10.1371/journal.pone.0291178)
Supplement: S1 Table — (DOCX) [file pone.0291178.s007.docx]

**S1 Table.** **Search strategy used in PubMed, Embase,** **and Web of science**

| **Database** | **Search number** | **Query** | **Results** | **Date** |
| --- | --- | --- | --- | --- |
| PubMed | #4 | (((COVID-19[Title/Abstract]) OR (sars-cov-2[Title/Abstract]) OR (coronavirus disease 2019[Title/Abstract]) OR (2019-nCoV[Title/Abstract]) AND ((cancer[Title/Abstract]) OR (neoplasms[Title/Abstract]) OR (carcinoma[Title/Abstract]) OR (malignancy[Title/Abstract]) AND ((lung[Title/Abstract]) OR (pulmonary[Title/Abstract]) | 1,834 | February 20, 2023 |
|  | #3 | (lung[Title/Abstract]) OR (pulmonary[Title/Abstract]) | 937,149 | February 20, 2023 |
|  | #2 | (cancer[Title/Abstract]) OR (neoplasms[Title/Abstract]) OR (carcinoma[Title/Abstract]) OR (malignancy[Title/Abstract]) | 2,389,910 | February 20, 2023 |
|  | #1 | (COVID-19[Title/Abstract]) OR (sars-cov-2[Title/Abstract]) OR (coronavirus disease 2019[Title/Abstract]) OR (2019-nCoV[Title/Abstract]) | 326,165 | February 20, 2023 |
| Embase | #4 | #1 AND #2 AND #3 | 2,879 | February 20, 2023 |
|  | #3 | 'lung':ab,ti OR 'pulmonary':ab,ti | 1,401,169 | February 20, 2023 |
|  | #2 | 'cancer':ab,ti OR 'neoplasms':ab,ti OR 'carcinoma':ab,ti OR ' malignancy ':ab,ti | 3,373,362 | February 20, 2023 |
|  | #1 | 'COVID-19':ab,ti OR 'sars-cov-2':ab,ti OR 'coronavirus disease 2019':ab,ti OR '2019-nCoV ':ab,ti | 335,105 | February 20, 2023 |
| Web of science | #4 | #1 AND #2 AND #3 | 2,595 | February 20, 2023 |
|  | #3 | (TI=(lung)) OR (AB=(lung)) OR (TI=(pulmonary)) OR (AB=(pulmonary)) | 1,680,500 | February 20, 2023 |
|  | #2 | (TI=(cancer)) OR (AB=(cancer)) OR (TI=(neoplasms)) OR (AB=(neoplasms)) OR (TI=(carcinoma)) OR (AB=(carcinoma)) OR (TI=(malignancy)) OR (AB=(malignancy)) | 3,989,607 | February 20, 2023 |
|  | #1 | (TI=(COVID-19)) OR (AB=(COVID-19)) OR (TI=(sars-cov-2)) OR (AB=(sars-cov-2)) OR (TI=(coronavirus disease 2019)) OR (AB=(coronavirus disease 2019)) OR (TI=(2019-nCoV)) OR (AB=(2019-nCoV)) | 430,205 | February 20, 2023 |
