# Supplementary material for: Risk factors for mortality among lung cancer patients with covid-19 infection: A systematic review and meta-analysis
Source: PLoS One. 2023 Sep 8;18(9):e0291178. doi: 10.1371/journal.pone.0291178 (PMC10490932; doi:10.1371/journal.pone.0291178)
Supplement: S2 Table — The maximal score for Newcastle-Ottawa scale is 9 stars: 4 stars for the selection process, 2 stars for comparability, and 3 stars for outcome. (DOCX) [file pone.0291178.s008.docx]

**S2 Table. Quality assessment of the included studies by the Newcastle-Ottawa Scale**

| **Author/Year** | **Region** | **Selection** | **Comparability** | **Outcome** | **Total star** |
| --- | --- | --- | --- | --- | --- |
| Beltramo et al., 2021 | France | **** | * | * | ****** |
| Bernard et al., 2021 | France | *** | * | ** | ****** |
| Chen et al., 2022 | USA | *** | * | ** | ****** |
| Provencio et al., 2021 | Spain | *** | * | * | ***** |
| Lièvre et al.,2020 | France | *** | ** | ** | ******* |
| Várnai et al.,2020 | UK | *** | * | ** | ****** |
| Haineala et al., 2021 | Romania | **** | ** | * | ******* |
| Farooque et al., 2021 | Pakistan | **** | ** | ** | ******** |
| Özdemir et al.,2020 | Turkey | **** | * | ** | ******* |
| Joode et al., 2021 | Netherlands | **** | * | *** | ******** |
| Peixoto et al., 2022 | Brazil | **** | ** | * | ******* |
| Luo et al., 2020 | USA | *** | ** | ** | ******* |
| Lee et al.,2020 | UK | **** | * | * | ****** |
| Benderra et al., 2021 | France | *** | * | * | ***** |
| Bursać et al., 2022 | Serbia | **** | * | ** | ******* |
| Song et al.,2021 | China | *** | * | ** | ****** |
| Nie et al., 2020 | China | **** | * | * | ****** |
| Dai et at., 2020 | China | *** | ** | *** | ******** |
| Basse et al., 2021 | France | **** | * | *** | ******** |
| Rogado et al., 2020 | Spain | **** | ** | * | ******* |
| Ferrari et al., 2020 | Brazil | *** | * | ** | ****** |
| Yarza et at., 2020 | Spain | **** | ** | * | ******* |
| Yang et al.,2021 | China | *** | * | *** | ******* |
| Khusid et al.,2021 | USA | *** | ** | * | ****** |
| Mehta et al.,2020 | USA | *** | * | *** | ******* |
| Stroppa et al.,2020 | Italy | *** | * | ** | ****** |
| Fraser et al., 2021 | UK | **** | * | * | ****** |
| de Melo et al., 2020 | Brazil | *** | * | *** | ******* |
| Hogan et al., 2020 | UK | *** | * | *** | ******* |

The maximal score for Newcastle-Ottawa Scale is 9 stars: 4 stars for the selection process, 2 stars for comparability, and 3 stars for outcome.
