# Supplementary material for: Risk factors for mortality among lung cancer patients with covid-19 infection: A systematic review and meta-analysis
Source: PLoS One. 2023 Sep 8;18(9):e0291178. doi: 10.1371/journal.pone.0291178 (PMC10490932; doi:10.1371/journal.pone.0291178)
Supplement: S3 Table — The maximal score for Prevalence Study Quality is 11, and only YES means 1 score. (DOCX) [file pone.0291178.s009.docx]

**S3 Table. Quality assessment of the included studies by the Prevalence Study Quality**

| **Item (YES/NO/UNCLEAR)** | **Author/Year** | | | |
| --- | --- | --- | --- | --- |
|  | **Kahya et al., 2021** | **Calles et al., 2020** | **Zhang et al., 2020** | **Fernandes et al., 2021** |
| 1) Define the source of information (survey, record review) | YES | YES | YES | YES |
| 2) List inclusion and exclusion criteria for exposed and unexposed subjects (cases and controls) or refer to previous publications | YES | YES | YES | YES |
| 3) Indicate time period used for identifying patients | YES | YES | YES | YES |
| 4) Indicate whether or not subjects were consecutive if not population-based | YES | NO | NO | NO |
| 5) Indicate if evaluators of subjective components of study were masked to other aspects of the status of the participants | NO | NO | NO | NO |
| 6) Describe any assessments undertaken for quality assurance purposes (e.g., test/retest of primary outcome measurements) | YES | NO | YES | NO |
| 7) Explain any patient exclusions from analysis | YES | YES | YES | YES |
| 8) Describe how confounding was assessed and/or controlled. | YES | YES | YES | YES |
| 9) If applicable, explain how missing data were handled in the analysis | UNCLEAR | UNCLEAR | UNCLEAR | UNCLEAR |
| 10) Summarize patient response rates and completeness of data collection | YES | YES | YES | YES |
| 11) Clarify what follow-up, if any, was expected and the percentage of patients for which incomplete data or follow-up was obtained | NO | NO | YES | YES |
| Total score | 8 | 6 | 8 | 7 |

The maximal score for Prevalence Study Quality is 11, and only *YES* means 1 score.
