# Supplementary material for: Risk factors for mortality among lung cancer patients with covid-19 infection: A systematic review and meta-analysis
Source: PLoS One. 2023 Sep 8;18(9):e0291178. doi: 10.1371/journal.pone.0291178 (PMC10490932; doi:10.1371/journal.pone.0291178)
Supplement: S4 Table — # variables were set as the reference. *P value < 0.05. (DOCX) [file pone.0291178.s010.docx]

**S4 Table. The result of meta-regression analysis when evaluating the pooled mortality of lung cancer in COVID-19 patients**

| **Subgroup** | **Variables** | **Estimate.** | ***Z*** | ***P*** |
| --- | --- | --- | --- | --- |
| Center type | Multi-center^#^ |  |  |  |
|  | Single | 0.039 | 0.595 | 0.552 |
| Continents | Asia^#^ |  |  |  |
|  | Europe | 0.258 | 4.926 | < 0.001* |
|  | North America | 0.102 | 1.340 | 0.180 |
|  | South America | 0.356 | 4.212 | < 0.001* |
| Publication year | Year | -0.051 | -1.112 | 0.266 |
| Study design | Prospective^#^ |  |  |  |
|  | Retrospective | 0.070 | 0.982 | 0.326 |
| Study type | Case control study^#^ |  |  |  |
|  | Cohort study | 0.197 | 2.227 | 0.056 |
|  | Cross-sectional study | 0.166 | 1.239 | 0.215 |
| Diagnosis methods | RT-PCR^#^ |  |  |  |
|  | RT-PCR/Antibodies | 0.077 | 0.456 | 0.648 |
|  | RT-PCR/CT | 0.033 | 0.163 | 0.871 |
|  | RT-PCR/CT/Clinical | 0.019 | 0.227 | 0.821 |

^#^ variables were set as the reference. **P* value < 0.05
